# Physicochemical analysis and biological characterization of FKB327 as a biosimilar to adalimumab

**DOI:** 10.1002/prp2.604

**Published:** 2020-06-04

**Authors:** Stefan Schreiber, Katsuhiko Yamamoto, Rafael Muniz, Takafumi Iwura

**Affiliations:** ^1^ Clinic for Internal Medicine Kiel Campus University Hospital Schleswig‐Holstein Kiel Germany; ^2^ Analytical Development Department Fujifilm Kyowa Kirin Biologics Co., Ltd. Tokyo Japan; ^3^ Global Medical Affairs Mylan Inc. Canonsburg PA USA; ^4^ Bio Process Research and Development Laboratories Production Division Kyowa Kirin Co., Ltd. Takasaki Japan

**Keywords:** adalimumab, biosimilar, Humira, monoclonal antibody, tumor necrosis factor

## Abstract

FKB327 was approved by the European Medicines Agency as a biosimilar to European‐authorized adalimumab (Humira^®^; AbbVie Inc). Adalimumab is a monoclonal antibody, binding and inhibiting tumor necrosis factor (TNF)‐α with use indicated for several immune‐mediated, chronic, and inflammatory disorders. The approval is based on high similarity in the physicochemical properties between FKB327 and adalimumab. The objective of this study is to assess the biological similarity, with regard to Fab‐ and Fc‐associated functions, and describe the relationship between physicochemical and biological characterization and functional activity. State‐of‐the‐art orthogonal techniques were implemented to assess the structure and function of FKB327. Peptide mapping with liquid chromatography and mass spectrometry, capillary electrophoresis–sodium dodecyl sulfate, ultraviolet circular dichroism, size‐exclusion high‐performance liquid chromatography (HPLC), and cation exchange HPLC were the techniques used to assess structure. Functional activity was assessed with enzyme‐linked immunosorbent assay, surface plasmon resonance, and cell‐based assays. The polypeptide sequence of FKB327 was identical to that of adalimumab. FKB327 also was demonstrated to have a similar secondary and tertiary structure to adalimumab. Posttranslational heterogeneities, along with size and charge variants, were not clinically meaningful. FKB327 binds to TNF‐α, FcγR, the neonatal Fc receptor, and C1q, and induces apoptosis, antibody‐dependent cellular cytotoxicity, and complement‐dependent cytotoxicity. The binding and activity of FKB327 were similar to that of adalimumab. FKB327 shares similar structure and activity with adalimumab. Based on characterization of physicochemical and biological properties, FKB327 is expected to have a similar safety, immunogenicity, and efficacy profile to adalimumab.

**Abbreviations**
ADCC: antibody‐dependent cellular cytotoxicity
CD: circular dichroism
CDC: complement‐dependent cytotoxicity
CE: capillary electrophoresis
CpB: carboxypeptidase B
DP: drug product
DS: drug substance
EC_50_: half maximal effective concentration
ELISA: enzyme‐linked immunosorbent assay
EMA: European Medicines Agency
FcRn: neonatal Fc receptor
FcγR: human Fc‐gamma receptor
FDA: US Food and Drug Administration
FFF: field‐flow fractionation
FITC: fluorescein isothiocyanate
FT‐IR: Fourier‐transform infrared
HC: heavy chain
HMWS: high‐molecular weight species
HPLC: high‐performance liquid chromatography
Ig: immunoglobulin
K_D_: equilibrium dissociation constant
LC: light chain
MS: mass spectrometry
PAGE: polyacrylamide gel electrophoresis
pI: isoelectric point
rh: recombinant human
RP: reference product
SDS: sodium dodecyl sulfate
SE: size‐exclusion
SPR: surface plasmon resonance
tm: transmembrane‐bound
TNF: tumor necrosis factor
UV: ultraviolet

## 1 INTRODUCTION

Biologics have become indispensable in the treatment of serious immunologic conditions, including chronic, immune‐mediated inflammatory diseases such as rheumatoid arthritis, Crohn's disease, psoriasis, and psoriatic arthritis.^1^ Antitumor necrosis factor (anti‐TNF) agents have the largest base of efficacy and safety data among all biologics. Anti‐TNF agents also have broad pediatric indications. In addition, documented clinical experience in pregnancy is now large enough that the black box warning for pregnancy has been lifted by the European Medicines Agency (EMA) and in other jurisdictions.^2^

Because biologics are produced in living systems, the manufacturing process for both reference products (RPs) and biosimilars is complex and cannot be exactly replicated.^3^ Differences in manufacturing can result in protein heterogeneity. Amino acid sequences of the proposed biosimilar drug should be identical with that of the RP; however, minor differences may exist in terminal amino acid sequences because biologics are produced in living systems.^4^ Potential differences between a biosimilar and the RP include posttranslational modifications, such as glyxosylation, oxidation, deamidation, and protein aggregation, which are also caused by different host cell and expression systems. The bioprocess from production to purification and formulation for long‐term storage should be assessed to determine the clinical impact on pharmacokinetics, efficacy, and safety. The surveillance of biosimilarity is therefore part of the production algorithm. Biosimilars are biological products in which a genetically identical protein molecule is produced using new production cells and reinvention of the manufacturing procedures. Biosimilars have to be highly similar to the licensed biologic RP in terms of analytical characterization, biological function, purity, and pharmacokinetics/pharmacodynamics.^5^ Both the EMA and the US Food and Drug Administration (FDA) have developed tight guidance for the development of biosimilars.^6^, ^7^ The FDA guidance recommends a totality‐of‐evidence approach.^7^ In the guidance for quality consideration for biosimilar development, an extensive analytical and functional similarity assessment is required to demonstrate that the biosimilar product has a highly similar quality profile with the RP. Therefore, sensitive and comprehensive side‐by‐side analyses of the biosimilar and RP using state‐of‐the‐art analytical technologies should be designed to determine similarities and potential differences in quality attributes so that the attributes of the biosimilar are appropriately assessed to determine the potential impact on safety and efficacy.^7^, ^8^ Evaluation occurs in a stepwise process, with structural and functional testing being the first and foundational step.^6^

The nonclinical development of FKB327 (Hulio^®^) was performed in accordance with the “Guideline on similar biological medicinal products containing monoclonal antibodies: non‐clinical and clinical issues”;^6^ with the “Guideline on similar biological medicinal products containing biotechnology‐derived proteins as active substance: non‐clinical and clinical issues”;^8^ and with ICH guideline S6 (R1)—preclinical safety evaluation of biotechnology‐derived pharmaceuticals.^9^

Fujifilm Kyowa Kirin Biologics has conducted physicochemical and biological analyses to assess the analytical similarity of FKB327 with EU‐approved Humira and with US‐licensed Humira. In each similarity assessment exercise, EU‐approved Humira or US‐licensed Humira was used as the reference product. These studies were conducted to demonstrate the biosimilarity of FKB327 with both EU‐approved Humira and with US‐licensed Humira. The studies have also provided bridging data between EU‐approved Humira and US‐licensed Humira to support use of US‐licensed Humira in the Phase 3 program.

FKB327 is a biosimilar to adalimumab (Humira^®^; AbbVie Inc). Adalimumab is an immunoglobulin (Ig)G1 monoclonal antibody that binds and inhibits TNF‐α.^2^ TNF‐α is a cytokine involved in the inflammatory response that has been shown to be overexpressed in autoimmune diseases. Adalimumab is indicated for the treatment of rheumatoid arthritis, juvenile idiopathic arthritis, psoriatic arthritis, ankylosing spondylitis, adult and pediatric Crohn's disease, ulcerative colitis, plaque psoriasis, hidradenitis suppurativa, and adult and pediatric uveitis. The EMA reports additional indications of axial spondyloarthritis and pediatric plaque psoriasis.^10^

For the work presented here, state‐of‐the‐art techniques were used to assess the structure and function of FKB327 in comparison with adalimumab to determine a high level of similarity. Based on physicochemical analysis and biological characterization, FKB327 should have similar clinical outcomes to both US‐licensed and EU‐approved adalimumab across all disease states for which adalimumab is approved.

## 2 MATERIALS AND METHODS

FKB327 drug substance (DS) was manufactured by Kyowa Kirin using the DS formulation buffer. During the analytical similarity assessment, FKB327 DS (~70 mg/mL) was used as the test product. FKB327 drug product (DP) (50 mg/mL) was manufactured by Terumo using the DP formulation buffer, which consisted of monosodium glutamate (10 mmol/L), sorbitol (262 mmol/L), polysorbate 80 (1.0 mg/mL), methionine (5 mmol/L), diluted hydrochloric acid (adjust to pH 5.2), and water. The FKB327 DS formulation is similar to the FKB327 DP formulation, with only methionine being added to the DP formulation buffer. FKB327 DP was used during the similarity assessment to assess strength, visible and subvisible particles, cytotoxicity neutralization, and TNF‐α binding activities, as well as to characterize the protein in cell‐based assays.

For the analyses, one lot each of FKB327, EU‐approved adalimumab, and US‐licensed adalimumab were used to assess primary structure, whereas 10 lots each were used to assess glycosylation, size and charge heterogeneity, amino acid modifications, process‐related impurities, and binding. For higher‐order structure, three lots each were used for the investigations. For visible and subvisible particle analysis, 22 lots of FKB327 and three lots of US‐ and EU‐approved adalimumab were used. To assess strength, 22 lots of FKB327, 26 lots of EU‐approved adalimumab, and 30 lots of US‐licensed adalimumab were used.

### 2.1 Physicochemical characterization

#### 2.1.1 Primary structure and posttranslational modifications

For peptide mapping, peptide mixtures were analyzed on a C18 reversed‐phase high‐performance liquid chromatography (HPLC), column coupled online to an ion trap electrospray mass spectrometer.^11^ The N‐terminal amino acid sequence of FKB327 was determined by automated Edman degradation sequencing.^12^ FKB327 and adalimumab were reduced and analyzed by sodium dodecyl sulfate (SDS)–polyacrylamide gel electrophoresis (PAGE) to separate into heavy chain (HC) and light chain (LC) molecules. The HC and the LC molecules were transferred to a polyvinylideine fluoride membrane by blotting,^13^ and then the HC and LC bands were examined by N‐terminal sequencing. As no variability is expected from this technique, only a single batch of each product was tested. The molecular weights of FKB327 DS and adalimumab were determined by electrospray ionization time‐of‐flight–mass spectrometry (MS).^14^ Isoelectric focusing was conducted using a polyacrylamide matrix with a gradient of pH 3‐10. The extinction coefficient was determined using the protein content measured by amino acid analysis and ultraviolet (UV) absorbance at 280 nm.

#### 2.1.2 Glycosylation

The N‐linked glycan profiles of FKB327 and adalimumab were evaluated by reversed‐phase HPLC. Each peak was fractionated and analyzed by matrix‐assisted laser desorption/ionization time‐of‐flight–MS to elucidate structures.^15^ The monosaccharide contents of FKB327 DS and adalimumab were determined by reversed‐phase HPLC after fluorescein labeling.

#### 2.1.3 High‐order structure

The secondary structures were examined using Fourier‐transform infrared (FT‐IR) spectroscopy.^16^ The secondary and tertiary conformations were examined by far‐ and near‐UV circular dichroism (CD) spectroscopy, respectively. The tertiary structures of FKB327 and adalimumab were examined by intrinsic fluorescence spectroscopy, where the fluorescence spectrum was determined at 280 nm and emission spectra were recorded in a range of 290‐450 nm. The measurement was conducted on both sample solutions, but the formulation buffer was replaced with formulation buffer (specific to each product) prepared with deuterium oxide.The thermal transition properties were examined by differential scanning calorimetry; the temperature range of the scan was 25‐100°C and the scan rate was set at 1°C/min.

#### 2.1.4 Size variants

The separation of size variants is based on an electromotive force to drive the molecules through a sieve‐like matrix of polyacrylamide gel, with a capillary electrophoresis (CE) system using a fused silica capillary after fluorescence labeling of these proteins under nonreducing and reducing conditions. Laser‐induced fluorescence detection was conducted with excitation at 488 nm and emission at 560 nm. The size heterogeneity of FKB327 and adalimumab was also evaluated using size‐exclusion (SE)‐HPLC. SE‐HPLC can separate the molecules based on their hydrodynamic radius. SE‐HPLC was performed using a Tosoh TSKgel G3000SWXL column (Tosoh Bioscience GmbH) with a mobile phase of 50 mmol/L sodium phosphate buffer containing 500 mmol/L sodium chloride and 5% (vol/vol) ethanol, at pH 7.0 and monitoring UV absorbance at 215 nm. The size heterogeneity of FKB327 and adalimuab was also evaluated using field‐flow fractionation (FFF) as an orthogonal test to SE‐HPLC. FFF analysis was conducted using a mobile phase of phosphate‐buffered saline (PBS). Detection was done by monitoring UV absorbance at 215 nm.

#### 2.1.5 Charge variants

A CE chromatography‐based HPLC method was used to separate charge variants in FKB327 and adalimumab with a Dionex™ ProPac™ WCX‐10 (Thermo Fisher Scientific) with a sodium chloride gradient in sodium phosphate buffer, pH 6.5. Eluted peaks were detected at 280 nm.

#### 2.1.6 Purity, strength, and stability

Process‐related impurities in the form of residual DNA content were measured using a threshold assay.^17^ The content of host‐cell protein was measured using a commercial enzyme‐linked immunosorbent assay (ELISA) kit (Cygnus Technologies). Visible particles were measured by visual inspection. The numbers of subvisible particles with sizes ≥2, ≥5, ≥10 and ≥25 μm were measured using the compendial light obscuration assay. Particles ≥5 μm were characterized using microflow imaging analysis.^18^

Protein concentration was determined by measuring the absorbance at 280 nm for the diluted sample solution, using the experimentally determined extinction coefficient of 1.4/mg/cm/mL. Stability studies under accelerated (25°C) and stressed (40°C) conditions were conducted to assess the degradation profiles.

### 2.2 Biological characterization

#### 2.2.1 Binding

The binding activity of FKB327 and adalimumab to soluble TNF‐α was assessed by ELISA in a range of 1‐4000 ng/mL. The binding affinity of FKB327 and adalimumab to recombinant human (rh)TNF‐α (0.75‐12 nmol/L) in a concentration of 0.25 μg/mL of FKB327 and transmembrane‐bound (tm)TNF‐α in a range of 0.03‐3000 ng/mL of FKB327 and adalimumab was determined using surface plasmon resonance (SPR) analysis and fluorescence‐activated cell‐sorting analysis, respectively. The SPR assay was conducted to determine binding affinity to human Fc‐gamma receptor (FcγR) and neonatal Fc receptor (FcRn). His‐tagged human FcγR I, IIa, IIb, IIIa(F), IIIa(V), IIIbNA1, IIIbNA2 or human FcRn was added to a BIAcore^TM^ biosensor chip (Biaffin GmbH & Co. KG) immobilized with anti‐His antibody for Fcγ receptors or anti‐β2‐microglobulin antibody for FcRn. FKB327 or adalimumab samples were injected over the surface of a biosensor chip and were captured by Fc receptors. FKB327 or adalimumab was diluted >5000‐fold by an assay buffer (Fcγ receptor RI and FcRn) or the formulation was exchanged by an assay buffer (Fcγ receptor RIIa, RIIb, RIIIa(F), RIIIa(V), RIIIbNA1, and RIIIbNA2) to eliminate a formulation effect. Various concentrations of FKB327 or adalimumab (0.125‐4.0 μg/mL for Fcγ receptor RI, 0.125‐4.0 mg/mL for Fcγ receptor RIIa, RIIb, RIIIa(F), RIIIa(V), RIIIbNA1, and RIIIbNA2; 0.15625‐2.5 μg/mL for FcRn) were injected over the surface of the biosensor. The equilibrium dissociation constant (K_D_) was calculated by nonlinear regression of the binding curves using 1:1 Langmuir binding model for FcγRI, a steady state affinity model for the other Fcγ receptors or a bivalent analyte model for FcRn. The equilibrium dissociation constant (K_D_) was calculated by nonlinear regression of the binding curves using a 1:1 Langmuir binding model (rhTNF‐α and FcγRI), a steady state affinity model (for other Fcγs), or a bivalent analyte model (FcRn). The binding activity to complement protein C1q was evaluated by ELISA. Human C1q solutions were added to microplates coated with various concentrations (0.156‐20 μg/mL) of FKB327 or adalimumab and the plates were incubated for 1 hour at 25°C. The wells were washed to remove unbound C1q and then C1q bound to FKB327 or adalimumab was detected with horseradish peroxidase‐labeled anti‐C1q antibody. Half maximal effective concentration (EC_50_) values that represent 50% of the maximal binding activity were determined, and the relative value was calculated from each EC_50_ value. The neutralizing activity for rhTNF‐α‐mediated cytotoxicity was determined using a cell‐based assay with an L929 cell line to demonstrate the potency of FKB327 DP or adalimumab. Various concentrations (0.06‐1000 ng/mL) of FKB327 or adalimumab were added to each well of the plate. The rhTNF‐α solution and cultured cells were then added to the well containing the test samples or the reference standard. After incubation for 22‐26 hours at 37°C in the presence of actinomycin D, intracellular adenosine triphosphate was measured as the index of viable cells.

#### 2.2.2 Induction of effector function

Effector function activity was evaluated using a cell‐based assay with a tmTNF‐α EL4 cell line. The degree of apoptosis induction was determined by flow cytometry with Annexin V‐ fluorescein isothiocyanate (FITC). Various concentrations (0.018‐40 μg/mL) of FKB327 or adalimumab were added to mTNF‐α/EL4 cells expressing mTNF‐α to induce apoptosis. After incubation for 19‐26 hours at 37°C, the degree of apoptosis induction was determined by flow cytometry with Annexin V‐FITC. The degree of antibody‐dependent cellular cytotoxicity (ADCC) and complement‐dependent cytotoxicity (CDC) was determined by fluorescence measurement. The target cells were incubated with varying concentrations of FKB327 or adalimumab (0.05‐100 ng/mL) and NK‐92 cells as an effector cell at 37°C to induce ADCC. After incubation for 1 hour at 37°C, the degree of cell death was determined by measurement of fluorescence on the supernatant of the reaction mixture. For determination of the relative activity to the FKB327 reference standard (RS), EC_50_ values that represent 50% of the maximal activity were determined for both the RS and test samples, and then the relative value was calculated from each EC50 value.

The CDC assay was conducted on FKB327 and adalimumab to investigate the similarity of the CDC activity. TNF‐α/EL4 cells, which are the target cells, were incubated with varying concentrations of FKB327 or adalimumab (1.64‐1000 ng/mL) at 37°C after uptake of calcein. Rat complement was added, followed by further incubation at 37°C to induce CDC. The degree of cell death was determined by measurement of fluorescence on the supernatant of the reaction mixture. For determination of the relative activity to the FKB327 RS, EC_50_ values that represent 50% of the maximal activity were determined for both the RS and test samples and then the relative value was calculated from each EC_50_ value.

## 3 RESULTS

### 3.1 Physicochemical characterization

#### 3.1.1 Primary structure and posttranslational modifications

With peptide mapping analysis, it was demonstrated that the polypeptide sequence of FKB327 was identical to adalimumab. Edman sequencing showed that the N‐terminal amino acid sequence of FKB327 was identical to that of adalimumab for both the HC (EVQLVESGGG) and LC (DIQMTQSPSS). FKB327 DS and adalimumab contained two kinds of HC N‐terminal variants of Glu^1^ and pGlu^1^. The level of each variant was similar between FKB327 DS and adalimumab. The C‐terminal amino acid of FKB327, which was verified by mass analysis, was consistent with that of adalimumab in both the HC (theoretical, 660.36 m/z; FKB327, 660.25 m/z; adalimumab, 660.27 m/z) and the LC (theoretical, 869.36 m/z; FKB327, 869.45 m/z; adalimumab, 869.44 m/z).

In terms of molecular weight, five major peaks were observed in the spectrum due to the heterogeneity of N‐linked glycan structures (Figure S1). The molecular weight for each peak in FKB327 DS was slightly different compared with the molecular weight of each peak of adalimumab. This is likely due to differences in the heterogeneity of the C‐terminal lysine and N‐linked glycans. Two major peaks were observed in the MS spectra, when FKB327 DS and adalimumab were treated with carboxypeptidase B (CpB) and N‐glycosidase (Figure S2). The molecular weights of FKB327 DS and adalimumab treated with CpB and N‐glycosidase were comparable.

The extinction coefficient, which depends on primary peptide structure, was 1.4/(mg/mL)/cm. This value was consistent with that of adalimumab. The deamidation and oxidation level for each species was similar between FKB327 DS and adalimumab. The only differences were observed in the levels of oxidation at the Met^256^ residue and deamidation/isomerization at the Asn^290^ residue on the HC when FKB327 DS was compared with US‐licensed adalimumab (Table 1). Similar results were also seen with EU‐approved adalimumab.

**Table 1:** Analytical similarity assessment results for physiochemical properties of FKB327 compared with EU‐approved and US‐licensed adalimumab^a^

	Category	Quality attribute (test method)		Acceptance criteria	Result	Similarity assessment
EU‐approved adalimumab	Primary structure	N‐terminal amino acid sequence		To be consistent with adalimumab	Consistent with adalimumab	Pass
		Amino acid sequence				
		C‐terminal amino acid				
		Disulfide bond				
		N‐glycosylation site				
		Molecular weight				
		pI				
		Extinction coefficient				
	Glycosylation	SA, %	(N‐linked glycan profiling)	0.0‐0.7	2.7‐3.9	The minor difference in content of specific glycosylation is considered not to be clinically meaningful.
		IP, %		8.5‐13.0	3.9‐4.6	
		M5, %		2.7‐6.3	1.3‐1.8	
		G1F0, %		0.0‐0.6	0.6‐1.1	
		G0F0, %		0.6‐1.5	3.4‐4.2	
		F0, %		0.9‐1.4	4.0‐5.0	
		G2F1, %		1.0‐3.0	1.4‐2.7	
		G1F1, %		13.5‐22.6	18.5‐26.0	
		G0F1, %		62.0‐73.4	58.3‐68.9	
		F1, %		85.5‐90.0	86.8‐88.8	
		Gal/N, mol/mol		0.18‐0.32 mol/mol	0.24‐0.35 mol/mol	
US‐licensed adalimumab	Primary structure	N‐terminal amino acid sequence		To be consistent with adalimumab	Consistent with adalimumab	Pass
		Amino acid sequence				
		C‐terminal amino acid				
		Disulfide bond				
		N‐glycosylation site				
		Molecular weight				
		pI				
		Extinction coefficient				
	Glycosylation	SA, %	(N‐linked glycan profiling)	0.0‐0.5	2.7‐3.9	The minor difference in content of specific glycosylation is considered not to be clinically meaningful.
		IP, %		6.5‐15.5	3.9‐4.6	
		M5, %		1.9‐7.0	1.3‐1.8	
		G1F0, %		0.0‐0.6	0.6‐1.1	
		G0F0, %		0.5‐1.4	3.4‐4.2	
		F0, %		0.9‐1.3	4.0‐5.0	
		G2F1, %		1.7‐2.2	1.4‐2.7	
		G1F1, %		15.5‐19.9	18.5‐26.0	
		G0F1, %		65.0‐71.1	58.3‐68.9	
		F1, %		82.9‐92.5	86.8‐88.8	
		Gal/N, mol/mol		0.22‐0.27 mol/mol	0.24‐0.35 mol/mol	

Abbreviation: pI, isoelectric point.

^a^ Only the main assessments are shown here. Table S2 details the full list of physicochemical assessments.

#### 3.1.2 Glycosylation

Peptide mapping with and without deglycosylation demonstrated that the N‐glycosylation site of FKB327 was consistent with that of adalimumab and occurs at Asn^301^. The glycosylation site occupancy and nonconsensus glycosylation, which was determined by CE‐SDS, was similar for FKB327 DS and adalimumab (Table 1). Regarding N‐linked glycan profiling, the results for FKB327 DS and adalimumab indicated that the main peaks were G1F1 and G0F1, which corresponded to the isoforms of asialo‐, biantennary‐, and core‐fucosylated complex structures containing one or zero galactose residues, respectively. The minor N‐linked glycans other than G2F1S2 and G2F0, seen in FKB327 DS, were also seen in adalimumab. The content of mannose, fucose, and N‐acetylgalactosamine were similar between FKB327 DS and adalimumab. In addition to the higher content of sialic acids in FKB327, the level of galactose residue was also higher than in adalimumab (Table 1).

#### 3.1.3 High‐order structure

The IgG1 molecule has 16 disulfide bridges consisting of nine types of linkages, which were all identified in nonreduced peptide mapping. Based on N‐terminal sequencing, FKB327 has a similar disulfide arrangement in the hinge region to adalimumab. The trisulfide formation level in the interchain disulfide linkage between the HC and the LC was evaluated by peptide mapping with MS detection. The trisulfide form was present in FKB327 DS and adalimumab, although the level was higher in FKB327 DS (Table 1).

CD and FT‐IR spectra for FKB327 DS were identical to those obtained for adalimumab. FT‐IR spectroscopy confirmed secondary structure, and the spectrum showed beta‐sheet bands at 1638 and 1689/cm (Figure S3). Far‐UV CD spectrum showed a negative maximum near 217 nm and also demonstrates that FKB327 DS has a secondary structure that contains primarily a beta sheet. Near‐UV CD spectrum evaluated tertiary structure and showed positive peaks at 260, 265, and 292 nm, which were attributable to aromatic amino acid residues (Figure S4).

Slight differences were observed for the intrinsic fluorescence and differential scanning calorimetry measurements. The intrinsic fluorescence spectrum confirmed tertiary structure and showed a positive maximum near 325 nm with a similar maximum wavelength, which was attributable to tryptophan residues and indicated that tryptophan residues were present in a similar environment to those in adalimumab. Fluorescence intensity was higher with FKB327 DS but was similar when formulated in adalimumab buffer. The thermogram for FKB327 DS showed two main endothermic transitions at 75 and 84°C, higher than those of adalimumab, which were 72 and 84°C; however, when formulated in the adalimumab formulation buffer, FKB327 exhibited the same thermogram transitions.

#### 3.1.4 Size variants

CE‐SDS analysis revealed that the percentage and retention time for most peaks were similar between FKB327 DS and adalimumab. SE‐HPLC analysis indicated that the percentages of each species in FKB327 DS were similar to those in adalimumab; however, the retention time of the high–molecular weight species (HMWS) peak for FKB327 DS was slightly different from that for adalimumab (Figure S5). To characterize the HMWS in detail, the isolated HMWS fractions from both FKB327 DS and adalimumab were subjected to SE‐HPLC–multiangle laser light scattering, SDS–polyacrylamide gel electrophoresis, SDS‐SE‐HPLC, far‐UV CD measurement, fluorescence spectroscopy, FcRn binding assay, and biological characterization. The HMWS in both FKB327 DS and adalimumab were shown to be mainly dimer species. During the SDS–PAGE analysis under reducing conditions, the migration distance of the band for the covalently associated species was similar between FKB327 DS and adalimumab; therefore, the HMWS exhibited the native conformation, similar to the main species. Differences in the dose response between the HMWS of FKB327 and adalimumab were observed in the apoptosis, ADCC, and CDC assays. FFF results were consistent with the results obtained by SE‐HPLC.

#### 3.1.5 Charge variants

In evaluation of the isoelectric point, five charge isoforms were determined by isoelectric focusing, with isoelectric points (pIs) varying between 8.0 and 9.3. The main isoform had a pI of 8.5. Similarly, a major band at a pI of 8.5 and several minor bands were observed in adalimumab in the same pI range as the bands for FKB327 DS. The band intensity of the acidic minor bands of FKB327 DS was stronger than that of adalimumab, whereas basic minor band intensity of FKB327 DS was weaker when compared with that of adalimumab.

Using CE HPLC, a main peak in adalimumab was also eluted at approximately 34 minutes, and acidic peaks and basic peaks were observed at the same range as FKB327 (Figure S6). The percentage of the acidic peaks of FKB327 DS was greater than that of adalimumab, whereas the amount of the basic species was lower. The acidic peaks are expected to contain molecules with sialylated glycans; it was shown that the abundance of sialylated glycans in FKB327 DS was greater than in adalimumab.

As determined by the CpB‐treated samples, one of the principal factors contributing to the difference of charge heterogeneity between FKB327 DS and adalimumab is the number of C‐terminal lysine residue Pro449 on the HC, which occurs more frequently with FKB327.

The data for the cytotoxicity neutralizing activity, apoptosis activity, ADCC activity, and CDC activity demonstrated that the biological activities of all fractions were similar between FKB327 DS and adalimumab.

#### 3.1.6 Purity, strength, and stability

Upon examination of host‐cell impurities, the residual DNA content was consistent across 10 lots of FKB327 DS at <2 pg/mg of protein, and the results were similar to those for adalimumab. The host‐cell protein contents, measured using ELISA, were consistent across 10 lots of FKB327 DS at <3 ng/mg of protein and were lower than those in adalimumab. Upon visual inspection, both FKB327 DP and adalimumab were practically free from visible particles. The numbers of subvisible particles with sizes ≥2, ≥5, ≥10, and ≥25 μm were fewer in FKB327 DP than in adalimumab.

The protein concentration of FKB327 DP was comparable to that of adalimumab and met the acceptance criterion. Both adalimumab and FKB327 DP contain the active substance adalimumab, the same dosage form, and the same administration route. The formulation is designed to provide appropriate osmolality and pH, and to prevent impurities.

Upon evaluation of stability, changes were seen for both FKB327 DP and adalimumab during accelerated and stressed conditions, and the rate of each change observed in FKB327 DP was similar to or lower than the rates observed for adalimumab. The long‐term stability study (2‐8°C) for three lots of adalimumab was conducted and confirmed no significant change in the quality of adalimumab, including protein concentration, potency, and subvisible particles during storage for the 24‐month shelf life.

### 3.2 Biological characterization

#### 3.2.1 Binding

**Figure 1:**
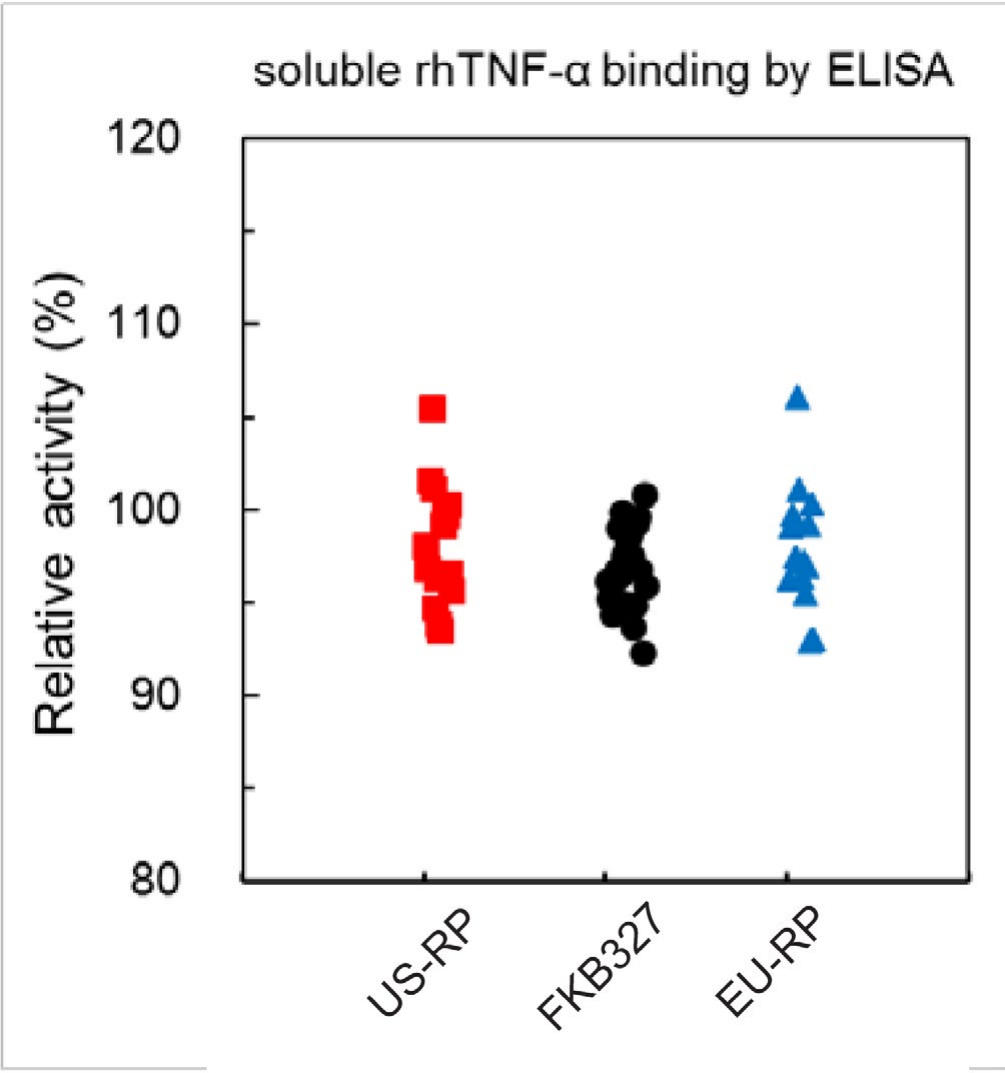
Relative binding activity of FKB327, US‐licensed adalimumab reference product, and EU‐approved adalimumab reference product to soluble TNF‐α. ELISA, enzyme‐linked immunosorbent assay; rh, recombinant human; RP, reference product (Humira^®^); TNF‐α, tumor necrosis factor alpha

**Figure 2:**
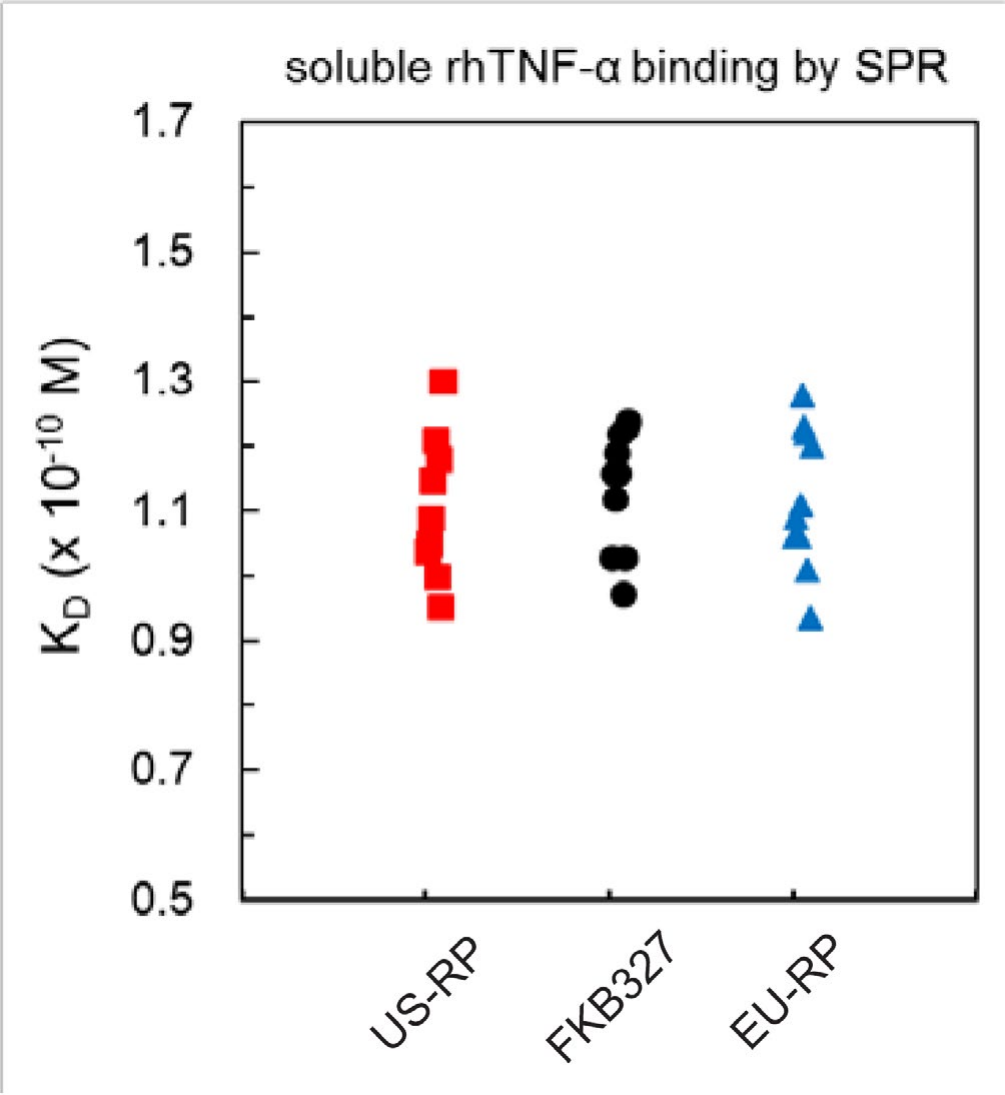
Binding affinity of FKB327, US‐licensed adalimumab reference product, and EU‐approved adalimumab reference product to soluble TNF‐α. K_D_, equilibrium dissociation constant; rh, recombinant human; RP, reference product (Humira^®^); SPR, surface plasmon resonance; TNF‐α, tumor necrosis factor alpha

The binding activity and affinity of FKB327 to rhTNF‐α was similar to that of adalimumab at DP level across an evaluation of 22 lots of FKB327 DP and 15 lots of adalimumab (Table 2; Figures 1 and 2). The K_D_ was 1.14 × 10^−10^ mol/L. The binding activity of FKB327 to tmTNF‐α was similar to that of adalimumab. The SPR analysis indicated that FKB327 had similar affinity to all FcγRs and FcRns with adalimumab (Table 2). Similar C1q binding activities were seen between FKB327 DS and US‐licensed adalimumab (Table 2). Similar results were seen with EU‐approved adalimumab.

**Table 2:** Analytical similarity assessment results for biological properties of FKB327 compared with EU‐approved and US‐licensed adalimumab

	Category	Quality attribute (test method)		Acceptance criteria	Result	Similarity assessment
EU‐approved adalimumab	Binding to target antigen(s)	Binding to soluble rhTNF‐α (ELISA)		85.2%‐110.9%	92.3%‐100.8%	Pass
		Binding to soluble rhTNF‐α (SPR)		0.64‐1.60 × 10^−10^ mol/L	0.97‐1.24 × 10^−10^ mol/L	
		Binding to tmTNF‐α		61.1%‐133.5%	88.6%‐107.0%	
	Binding to Fc receptors	Binding to FcγR	I	2.46‐6.35 × 10^−10^ mol/L	3.82‐4.36 × 10^−10^ mol/L	Pass
			IIa	6.78‐9.62 × 10^−6^ mol/L	7.65‐8.47 × 10^−6^ mol/L	
			IIb	1.23‐1.91 × 10^−5^ mol/L	1.38‐1.57 × 10^−5^ mol/L	
			IIIa(F)	0.93‐1.09 × 10^−6^ mol/L	0.94‐1.00 × 10^−6^ mol/L	
			IIIa(V)	4.63‐5.41 × 10^−6^ mol/L	4.74‐5.05 × 10^−6^ mol/L	
			IIIbNA1	0.86‐1.39 × 10^−5^ mol/L	0.94‐1.09 × 10^−5^ mol/L	
			IIIbNA2	0.80‐1.21 × 10^−5^ mol/L	0.87‐0.99 × 10^−5^ mol/L	
		Binding to FcRn		2.74‐12.10 × 10^−8^ mol/L	6.64‐7.59 × 10^−8^ mol/L	
	Fab‐associated functions	Cytotoxicity neutralization		88.4%‐111.9%	91.1%‐106.4%	Pass
		Apoptosis		76.8%‐121.1%	94.1%‐105.4%	
	Fc‐associated functions	ADCC		69.5%‐130.9%	90.4%‐122.0%	Pass
		CDC		72.2%‐122.7%	91.3%‐101.4%	
	Binding to complements	Binding to C1q		79.1%‐105.2%	93.1%‐101.5%	Pass
US‐licensed adalimumab	Binding to target antigen(s)	Binding to soluble rhTNF‐α (ELISA)		85.4%‐111.0%	92.3%‐100.8%	Pass
		Binding to soluble rhTNF‐α (SPR)		0.59‐1.66 × 10^−10^ mol/L	0.97‐1.24 × 10^−10^ mol/L	
		Binding to tmTNF‐α		70.3%‐124.7%	88.6%‐107.0%	
	Binding to Fc receptors	Binding to FcγR	I	2.51‐5.74 × 10^−10^ mol/L	3.82‐4.36 × 10^−10^ mol/L	Similarity in binding to FcγR, RII, RIIIa(V), RIIIbNA1, and FcRn is confirmed. As for binding to RIIIa(F) and RIIIbNA2, it is also comparable although slightly out of the acceptance criterion.
			IIa	6.99‐9.57 × 10^−6^ mol/L	7.65‐8.47 × 10^−6^ mol/L	
			IIb	1.29‐1.93 × 10^−5^ mol/L	1.38‐1.57 × 10^−5^ mol/L	
			IIIa(F)	0.95‐1.08 × 10^−6^ mol/L	0.94‐1.00 × 10^−6^ mol/L	
			IIIa(V)	4.72‐5.32 × 10^−6^ mol/L	4.74‐5.05 × 10^−6^ mol/L	
			IIIbNA1	0.85‐1.36 × 10^−5^ mol/L	0.94‐1.09 × 10^−5^ mol/L	
			IIIbNA2	0.90‐1.07 × 10^−5^ mol/L	0.87‐0.99 × 10^−5^ mol/L	
		Binding to FcRn		5.13‐9.52 × 10^−8^ mol/L	6.64‐7.59 × 10^−8^ mol/L	
	Fab‐associated functions	Cytotoxicity neutralization		88.5%‐114.2%	91.1%‐106.4%	Pass
		Apoptosis		78.4%‐112.5%	94.1%‐105.4%	Pass
	Fc‐associated functions	ADCC		78.4%‐139.4%	90.4%‐122.0%	Pass
		CDC		86.0%‐111.1%	91.3%‐101.4%	
	Binding to complements	Binding to C1q		76.4%‐103.2%	93.1%‐101.5%	Pass

Abbreviations: ADCC, antibody‐dependent cellular cytotoxicity; CDC, complement‐dependent cytotoxicity; ELISA, enzyme‐linked immunosorbent assay; FcRn, neonatal Fc receptor; FcγR, Fc‐gamma receptor; rhTNF‐α, recombinant human tumor necrosis factor alpha; SPR, surface plasmon resonance; tmTNF‐α, transmembrane‐bound tumor necrosis factor alpha.

#### 3.2.2 Induction of effector function

**Figure 3:**
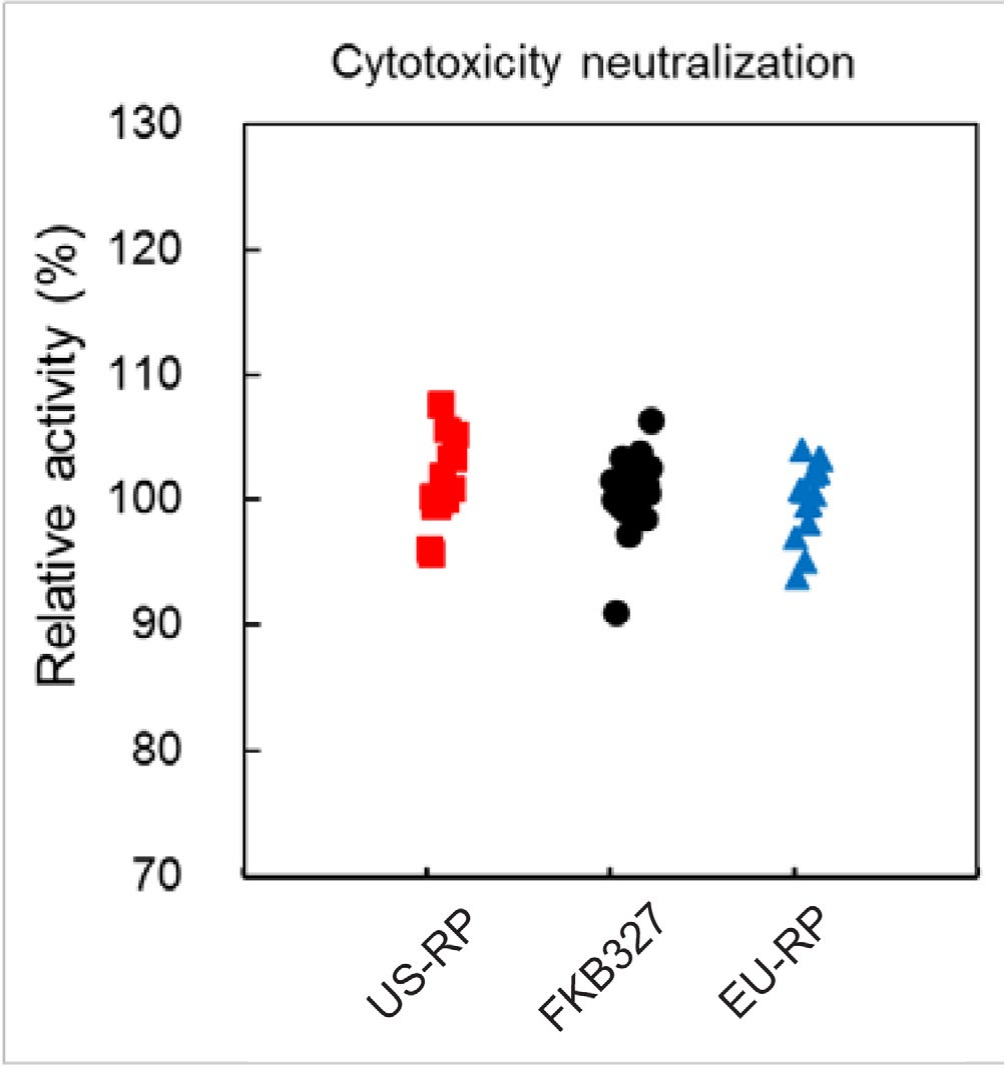
Relative cytotoxicity neutralization activity of FKB327, US‐licensed adalimumab reference product, and EU‐approved adalimumab reference product. RP, reference product (Humira^®^)

The cytotoxicity‐neutralizing activity of FKB327 DP was similar to that of adalimumab (Table 2; Figure 3). Similar apoptosis‐inducing activities were seen between FKB327 DP and adalimumab (Table 2). Similar ADCC activities were seen between FKB327 DP and adalimumab (Table 2). Similar CDC activities were seen between FKB327 DP and adalimumab (Table 2).

## 4 DISCUSSION

In this study, a wide battery of analytical and functional testing was used to characterize the structure and activity of FKB327 in an effort to demonstrate biosimilarity with adalimumab. Biosimilar guidance documents for quality assessment recommend stepwise assessment of the analytical and functional similarity of quality attributes based on the potential clinical impact.^7^, ^8^ That is, attributes that are potentially influenced by the drug product manufacturing process (eg strength) were assessed using more than 20 drug product lots each of FKB327, EU‐approved adalimumab, and US‐licensed adalimumab in nonclinical and clinical studies. Attributes that are potentially influenced by the drug substance bioprocess (eg glycans, size and charge heterogeneity, amino acid modifications, process‐related impurities, binding) were assessed using 10 representative lots manufactured from each drug substance. Primary and higher‐order structures were confirmed using 1‐3 lots of each drug substance manufactured by clinical scale.

A relationship has been demonstrated among protein structure, function, pharmacokinetics, and immunogenicity such that an identical structure is a prerequisite for similar function, similar pharmacokinetics, and similar immunogenicity.^19^ The polypeptide sequence was identical between FKB327 and adalimumab. The primary structure of a protein is the foundation of the protein's higher‐order structure and function. FKB327 and adalimumab shared the same pI and extinction coefficient, which are both representative of the primary protein structure. The secondary and tertiary structures showed a high degree of similarity between FKB327 and adalimumab, as predicted by the identical polypeptide sequence. Importantly, similar results were obtained using both the US‐licensed and EU‐approved adalimumab.

Peptide mapping identified only a few differences that did not meet acceptance criteria in posttranslational modifications between FKB327 and adalimumab; however, the differences are not expected to be clinically meaningful. The level of amidated Pro^449^, a C‐terminal variant, was higher in FKB327 than in adalimumab. The C‐terminal lysine variants are not expected to affect efficacy or safety based on the similar processing that occurs in natural human antibodies.^20^ The species with amidated Pro449 are also expected to have no significant effect on efficacy or safety, because the charge profile of amidated species was similar to the well‐characterized C‐terminal lysine variants.^21^ Amidated structures have been identified in antibody pharmaceuticals.^22^

The level of trisulfide form was higher in FKB327 than in the US‐licensed and EU‐approved adalimumab. Trisulfide form was a minor component for both FKB327 and adalimumab. It has been suggested that the trisulfide form has no significant impact on the antigen‐binding affinity in a number of antibodies and that the trisulfide form could be converted to the disulfide form during blood circulation after administration.^23^ The difference in the trisulfide content will not impact the safety and efficacy of the product. Overall, the levels of oxidation and deamidation are similar between FKB327 and adalimumab; however, the levels of oxidation at the Met^256^ residue and deamidation/isomerization at the Asn^290^ residue on the HC did not meet the respective acceptance criteria when FKB327 was compared with US‐licensed adalimumab. Oxidation and deamidation have been associated with increased immunogenicity.^24^ The differences were minor, and therefore, should not be considered clinically meaningful.

As with posttranslational amino acid modifications, only a few differences in N‐glycan structure were identified with N‐glycan profiling. FKB327 had a higher content of sialylated glycans than the US‐licensed and EU‐approved adalimumab. Terminal sialylation can increase the serum half‐life of many glycoproteins, and an increase in sialylation of the Fc glycan results in a decrease in ADCC activity.^25^ This, in the case of cell‐depleting drugs like rituximab, would be counterintuitive in contrast to anti‐TNF molecules where cellular toxicity is not intended. No studies to date demonstrate that antibody half‐life is affected by the content of sialylated glycans. When ADCC activity was evaluated using physiologic conditions, no impact on ADCC was reported despite the difference in fucosylation, suggesting that this difference is not of clinical importance.^26^ Therefore, the current biological similarity assessment indicates that the differences in the content of sialylated glycans between FKB327 and adalimumab do not contribute to differences in ADCC activity.

FKB327 had a higher content of afucosylated complex‐type glycans (F0) than adalimumab. Afucosylated complex‐type glycans can increase ADCC activity compared with fucosylated variants.^27^ The ADCC activity of FKB327 was shown to be similar to that of adalimumab, indicating that the difference in the glycosylation had no impact on ADCC activity. FKB327 had a higher content of Ga1 residues than adalimumab. Terminal Ga1 residues have a potential effect on CDC activity.^14^ No difference was seen between FKB327 and adalimumab in the results of the CDC analysis. G2F0 and G2F1S2 were detected only in FKB327. G2F0 and G2F1S2 are known as oligosaccharides synthesized in the human body. High‐mannose glycans can decrease serum half‐life;^28^ notably, the levels of high‐mannose glycans are lower in FKB327 than with adalimumab.

FKB327 has the same level of HMWS as adalimumab; however, both were overwhelmingly made up of monomers at a level of >99%. Differences in the dose response between the HMWS of FKB327 and adalimumab were observed in the apoptosis, ADCC, and CDC assays. Considering that the HMWS contents are very low for both FKB327 and adalimumab and these products currently show similar biological activity, the differences in the activities of HMWS from both products should not affect the efficacy of the product. HMWS can increase immunogenicity; however, it is thought no significant difference in immunogenicity should exist between FKB327 and adalimumab, because the HMWS exhibit the native conformation.^29^

It is important to remember that a biosimilar is a biologic product that is highly similar, but not identical to a licensed RP, such that there are “no clinically meaningful differences between the biologic product and the [RP] in terms of safety, purity, and potency,” which allows for minor differences in inactive components.^7^ Small differences between biosimilars and the RP are already expected due to manufacturing processes observed in different countries and by different companies. This is due, in part, to the complex manufacturing process for biologic products, which involves multiple stages.^30^ In addition, to ensure the integrity and stability of the product, the finished biologic drug is packaged, stored, and distributed under optimal environmental conditions.^4^, ^31^

FKB327 is currently available in Europe, and biosimilars to other RPs, including the infliximab biosimilar CT‐P13, are approved and available both in Europe and in the United States. No meaningful clinical differences have been reported to date. CT‐P13 also demonstrated high levels of similarity to infliximab.^32^ Differences in the native isoform MS profile were seen between CT‐P13 and infliximab, which were expected based on different glycosylation patterns. No important modifications were noted over time. Importantly, a study including 3112 patients with ulcerative colitis demonstrated that CT‐P13 was equivalent to infliximab, and the data suggested that the risk for serious infections was lower with CT‐P13 compared with infliximab in these patients.^33^

The trastuzumab biosimilar HLX02 has been demonstrated to be similar to Herceptin via the use of sensitive and orthogonal methods.^34^ In this study, high analytical similarity of HLX02 was demonstrated to both EU‐approved trastuzumab and China‐sourced trastuzumab. A novel FcγRIIIa affinity chromatography technique was used to quantitatively compare glycan effects on effector function, which demonstrated that HLX02 was more similar to RPs of the high FcγRIIIa affinity group.

An analysis of 31 bridging studies for 11 biosimilars, including adalimumab and infliximab, demonstrated that reference biological products originating from the European Union and the United States have nearly indistinguishable pharmacokinetic and pharmacodynamic properties.^35^ These findings suggest that additional in vivo bridging studies between RPs originating from different countries may not be necessary following in vitro study results demonstrating similar physicochemical and structural properties. Based on these reports, future approval of biosimilars may be based solely on physicochemical and biologic similarity demonstrated in 1 country, without the need for lengthy and expensive clinical trials and bridging studies. Safety is an important issue; however, this may only be accurately determined following several years of use in humans.

Despite the few physicochemical differences observed between FKB327 and adalimumab, FKB327 demonstrated similar Fab‐ and Fc‐binding activity, as well as similar induction of effector functions. The differences between FKB327 and adalimumab should not affect efficacy and safety, and the similarities predict that FKB327 should have similar clinical activity to adalimumab; these findings are predictive of clinical studies that have been conducted, including half‐life, immunogenicity, safety, and efficacy, which confirm that FKB327 is a biosimilar to adalimumab.

## 5 CONCLUSION

FKB327 shares a similar structure and function to that of US‐licensed and EU‐approved adalimumab, establishing biosimilarity between FKB327 and adalimumab. Importantly, there is a relationship between physicochemical and biological characteristics, as well as between properties and functional activity. Based on the findings of biosimilarity in structure and function between FKB327 and adalimumab, additional in vivo measures of pharmacokinetics and immunogenicity are likely to exhibit biosimilarity. Small differences identified between FKB327 and adalimumab should not impact immunogenicity, safety, or efficacy, but additional clinical investigations will evaluate these parameters. Based on physicochemical analysis and biological characterization, FKB327 should have similar clinical outcomes as both US‐licensed and EU‐approved adalimumab across all disease states for which adalimumab is approved.

## CONFLICT OF INTEREST

Stefan Schreiber reports personal fees outside the submitted work for advisory board consulting for Abbvie, Arena, Bristol‐Myers Squibb, Biogen, Celltrion, Celgene, I‐Mab, Gilead, MSD, Mylan, Pfizer, Fresenius, Janssen, Takeda, Theravance, Provention Bio, Protagonist, and Falk. Katsuhiko Yamamoto is an employee of Fujifilm Kyowa Kirin Biologics Co., Ltd. Rafael Muniz is an employee and shareholder of Mylan Inc. Takafumi Iwura is an employee of Kyowa Kirin Co., Ltd.

## ETHIC STATEMENT

This article is based on a preclinical study and does not have human participants or animals.

## AUTHOR CONTRIBUTIONS

SS, KY and TI: Conceptualization and Investigation. KY and TI: Data curation, Methodology, Project administration, Supervision, and Validation. KY, RM and TI: Formal analysis. KY: Funding acquisition, Resources and Software. SS: Writing – original draft. SS, KY, RM and TI: Writing–review and editing. All authors were fully responsible for all content and editorial decisions, and received no financial support or other form of compensation related to the development of this manuscript. All authors contributed to the interpretation of the results and critical revision of the manuscript for important intellectual content, approved the final manuscript, and take responsibility for the integrity of the data and accuracy of the data analysis.

## Supporting information

Supplementary Material

## Data Availability

The data that support the findings of this study are available from the corresponding author upon reasonable request.
