# Supplementary material for: Physicochemical analysis and biological characterization of FKB327 as a biosimilar to adalimumab
Source: Pharmacol Res Perspect. 2020 Jun 4;8(3):e00604. doi: 10.1002/prp2.604 (PMC7272391; doi:10.1002/prp2.604)
Supplement: Supplementary file 1 — Supplementary Material [file PRP2-8-e00604-s001.docx]

*Pharmacology Research & Perspectives*

**Physicochemical Analysis and Biological Characterization of an Adalimumab Biosimilar, FKB327, and the US- and EU-Approved Adalimumab Reference Product^[[1]](#footnote-1)^***

Stefan Schreiber, Katsuhiko Yamamoto, Rafael Muniz, Takafumi Iwura

**Figure S1. Deconvoluted Mass Spectra for Intact FKB327, US-Licensed Adalimumab Reference Product, and EU-Approved Adalimumab Reference Product**

RP indicates reference product.

**Figure S2. Deconvoluted Mass Spectra for Carboxypeptidase-B– and N-Glycosidase F Treated FKB327 Drug Substance, US-Licensed Adalimumab RP, and EU-Approved Adalimumab Reference Product**


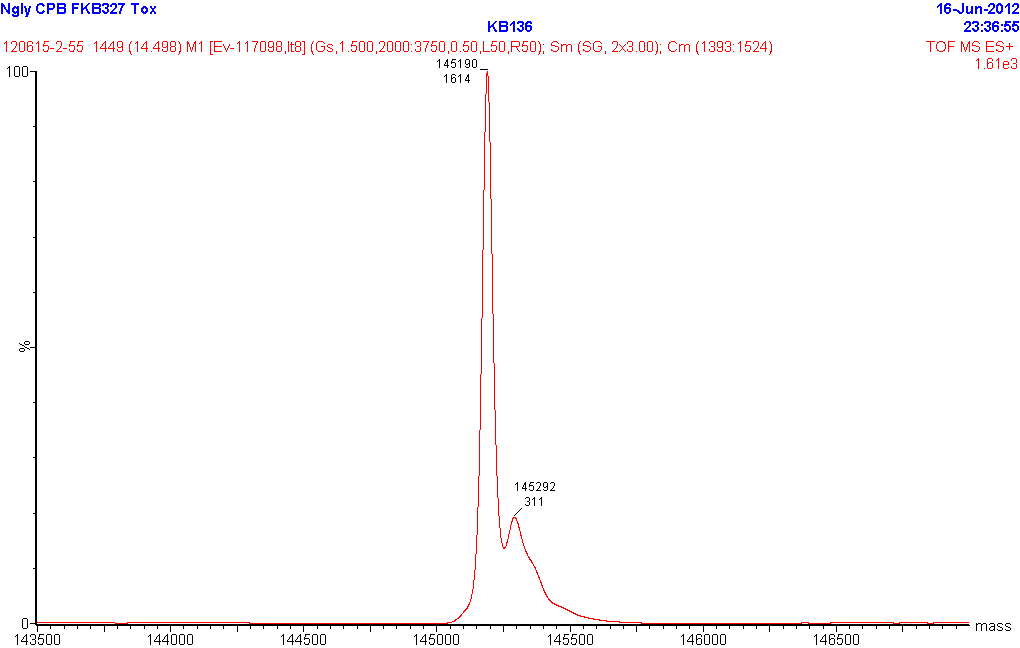


2

1

145190

145292

100

%

143500

144000

144500

145000

145500

146000

146500

FKB327


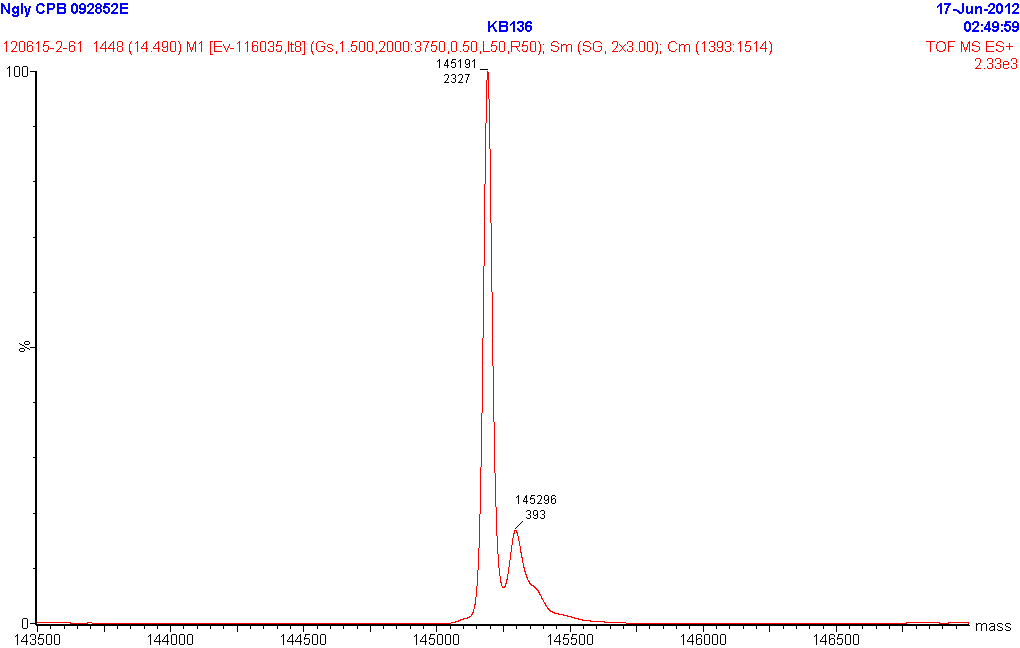


1’

2’

100

US-RP

145191

%

145296

144000

144500

145000

145500

146500

146000

143500


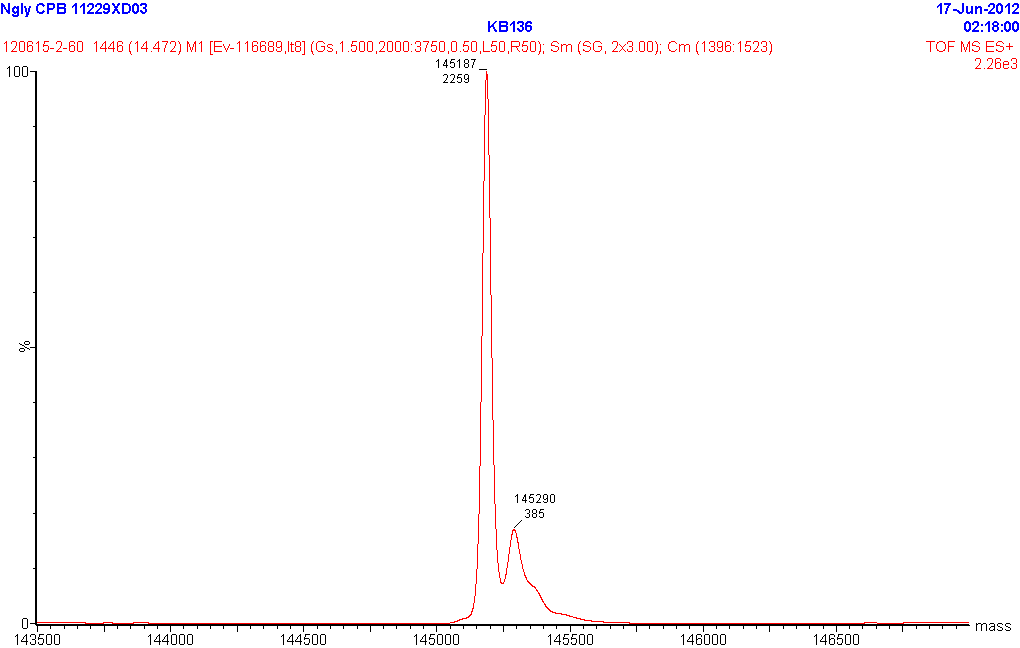


1’

2’

145187

145290

100

%

143500

144000

144500

145000

145500

146000

146500

EU-RP

RP indicates reference product.

**Figure S3. Second Derivative Fourier-Transform Infrared Spectra for FKB327 and US-Licensed Adalimumab Reference Product**


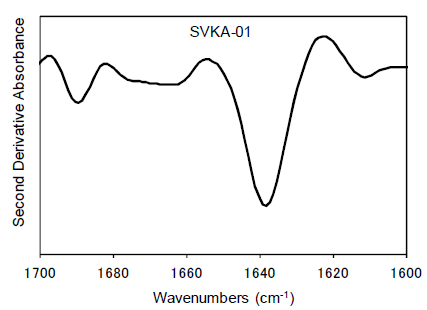


Second derivative absorbance

FKB327


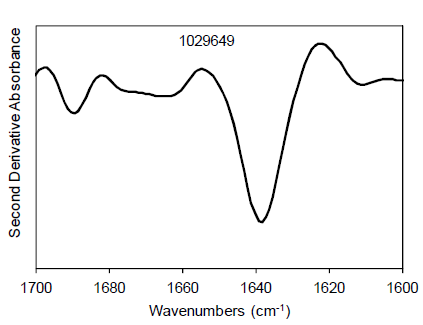


Second derivative absorbance

US RP


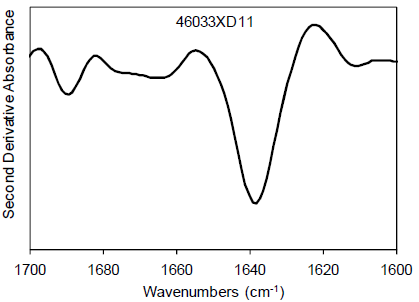


Second derivative absorbance

EU RP

RP indicates reference product.

**Figure S4. Near-Ultraviolet Circular Dichroism Spectra for FKB327, US-Licensed Adalimumab Reference Product, and EU-Approved Adalimumab Reference Product**


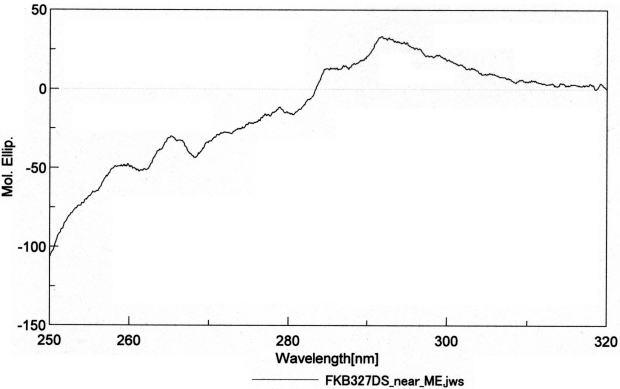


Mol. ellip.

FKB327

Wavelength [nm]


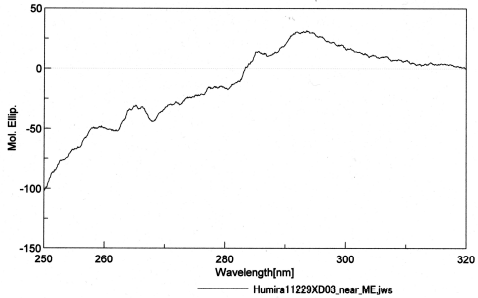


Mol. ellip.

EU RP

Wavelength [nm]


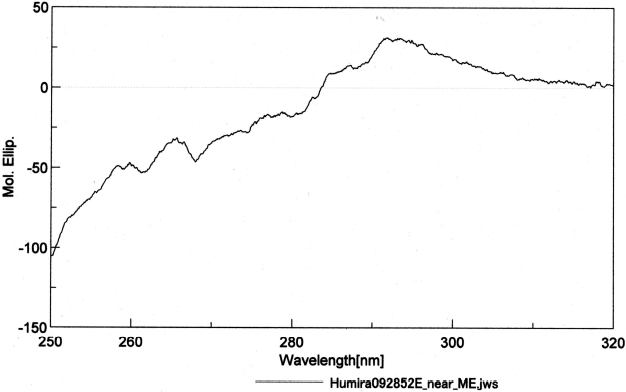


Mol. ellip.

US RP

Wavelength [nm]

RP indicates reference product.

**Figure S5. Size-Exclusion High-Performance Liquid Chromatography Chromatograms for FKB327 Drug Substance, US-Licensed Adalimumab Reference Product, and EU-Approved Adalimumab Reference Product**


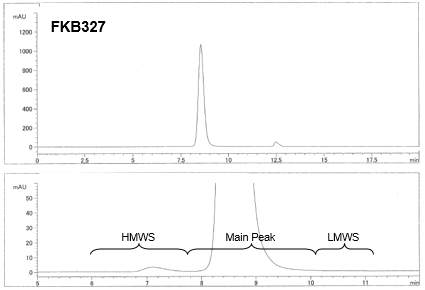


**EU RP**

**
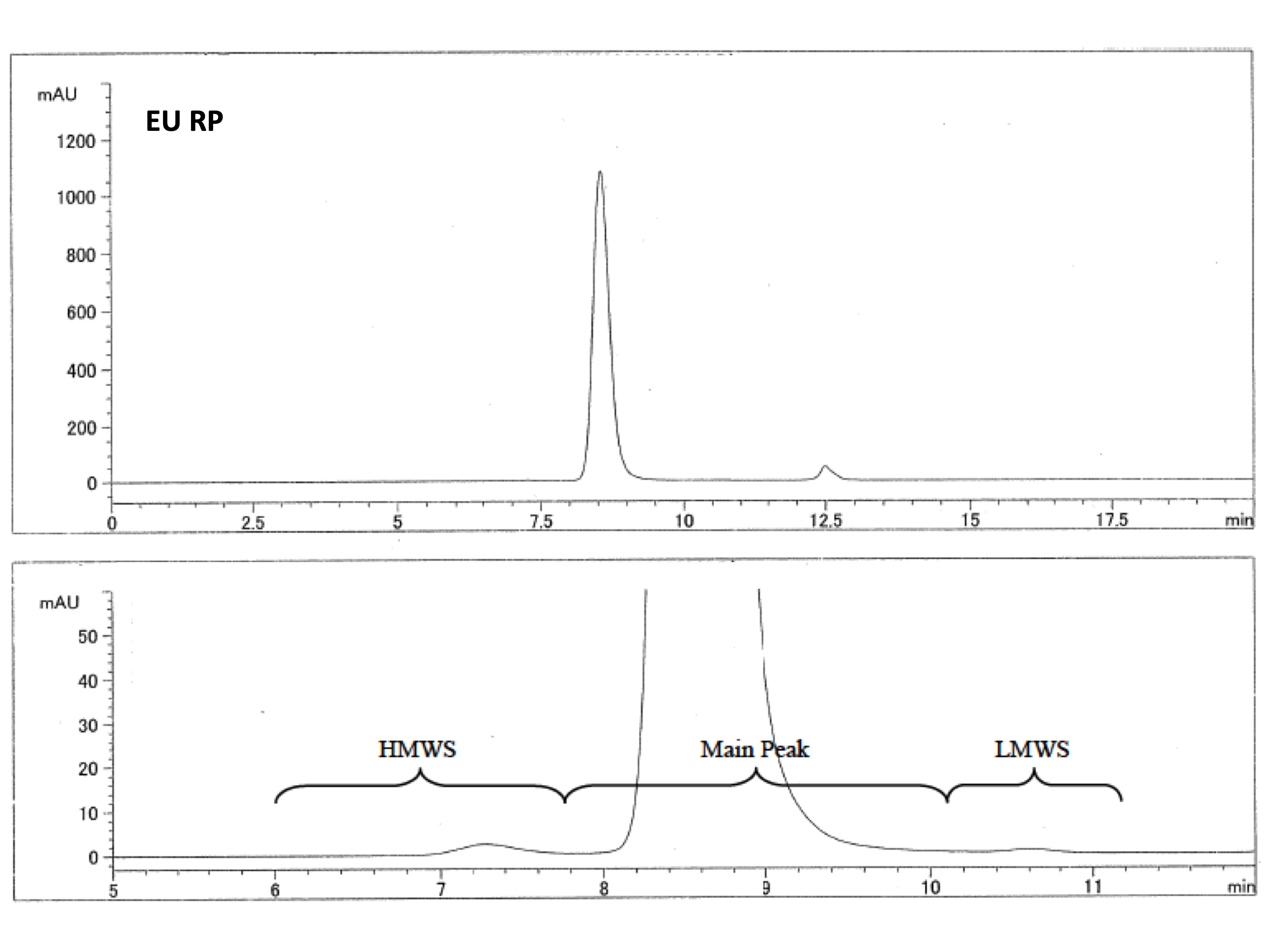
**

**US RP**


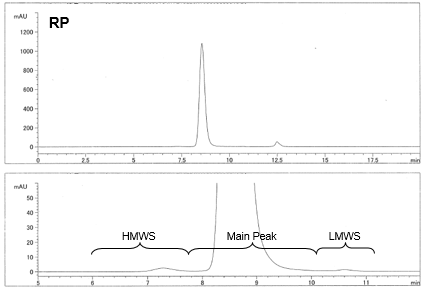


**Figure S6. Cation Exchange High-Performance Liquid Chromatography Chromatograms for FKB327 Drug Substance, US-Licensed Adalimumab Reference Product, and EU-Approved Adalimumab Reference Product**

FKB327

Acidic Peak

Basic Peak

Main Peak

US RP

Acidic Peak

Basic Peak

Main Peak

EU RP

Acidic Peak

Basic Peak

Main Peak

HMWS indicates high–molecular weight species; LMWS, low–molecular weight species; RP,

reference product (Humira^®^).

RP indicates reference product (Humira^®^)

1. [↑](#footnote-ref-1)
